# Idiopathic Raynaud’s Phenomenon and Vitamin D Deficiency in Children and Adolescents: An Exploratory Single-Center Retrospective Observational Study Throughout the Period 2010–2025

**DOI:** 10.3390/jcm15176516

**Published:** 2026-08-23

**Authors:** Donato Rigante, Michele Fastiggi, Cristina Guerriero, Marcello Candelli

**Affiliations:** 1 Department of Life Sciences and Public Health, Fondazione Policlinico Universitario A. Gemelli IRCCS, 00168 Rome, Italy; 2 Università Cattolica Sacro Cuore, 00168 Rome, Italy; 3 Pediatric Rheumatology Unit, Azienda USL-IRCCS di Reggio Emilia, 42123 Reggio Emilia, Italy; 4 Department of Dermatology, Fondazione Policlinico Universitario A. Gemelli IRCCS, 00168 Rome, Italy; 5 Department of Emergency Anesthesiological and Reanimation Sciences, Fondazione Policlinico Universitario A. Gemelli IRCCS, 00168 Rome, Italy; marcello.candelli@policlinicogemelli.it

**Keywords:** Raynaud’s phenomenon, 25-hydroxy-vitamin D, connective tissue disease, nailfold videocapillaroscopy, child, personalized medicine

## Abstract

***Background:*** Raynaud’s phenomenon (RP) may occur in the pediatric age span, sometimes representing the forerunner of devious underlying conditions. ***Objective:*** To investigate the prevalence of vitamin D deficiency in a single-center cohort of children and adolescents with idiopathic RP and evaluate changes in RP severity following cholecalciferol supplementation. ***Patients and Methods:*** Sixty-one children and adolescents with RP regularly followed at the pediatric rheumatology unit of our University between 2010 and 2025 were retrospectively evaluated. Clinical, laboratory, and nailfold videocapillaroscopy (NVC) findings were collected at baseline; patients were periodically examined for a mean investigation period of 3 ± 1.6 years. RP severity was assessed using the 2-week Raynaud’s Condition Score (RCS). Patients found to have serum 25-hydroxyvitamin D levels < 20 ng/mL received cholecalciferol supplementation for at least 12 weeks. ***Results:*** Nineteen out of 61 patients with RP (31.1% of the cohort) had vitamin D deficiency, while other laboratory parameters including inflammatory markers, complement fractions, liver and renal function tests, lipid profile, and autoimmune panel were non-informative. Univariate analysis demonstrated significant differences between vitamin D-deficient and non-deficient patients in terms of mean RP duration (*p* = 0.0002), baseline 2-week RCS (*p* = 0.0001), lupus-like anticoagulant positivity (*p* < 0.001), NVC pattern (*p* < 0.001), pain visual analogue scale (VAS) (*p* = 0.0001), and global quality of life VAS (*p* = 0.0001). After adjustment for age and sex, baseline 2-week RCS remained significantly associated with vitamin D deficiency. In addition, RCS significantly decreased during follow-up among vitamin D-deficient patients who received supplementation of cholecalciferol. ***Conclusions:*** Vitamin D deficiency may be common in pediatric idiopathic RP and pinpoint its severity: our exploratory study supports the routine assessment of vitamin D in children and adolescents with severe forms of RP, warranting prospective randomized controlled trials to determine whether correction of vitamin D deficiency may potentially improve disease severity.

## 1. Introduction

Raynaud’s phenomenon (RP) is an episodic vasospastic response to cold and/or emotional stimuli which is revealed by discoloration of the finger(s) or the toe(s) due to a reduction in the blood supply within the involved tissues. Patients classically experience a triphasic color change of fingers or toes: white/pallor, blue/cyanosis, and red/hyperemia [1]. The overall long-term prognosis of RP specifically depends on its relationship with eventual underlying illnesses [2]; however, this symptom, affecting around 3–5% of the general population, is largely idiopathic, i.e., without a frank explanatory cause. Frequently, RP is evaluated by pediatric rheumatologists because it may herald a connective tissue autoimmune disease, mostly systemic sclerosis [3]. Patients can report pain, tingling, or numbness impacting quality of life during attacks of RP [4]. Also, children and adolescents with RP must undergo a thorough investigation with close follow-ups to assess eventual underlying conditions, which subsequently may explain the phenomenon [5]. It remains challenging to define and objectively quantify the severity of RP in all ages and then implement a response to treatment through self-reported measures. Current therapies for RP may return ineffective results, though phosphodiesterase inhibitors and calcium channel blockers have shown efficacy in severe cases [6].

In our study we have evaluated clinical data, laboratory parameters and instrumental results of a single-center cohort of children and adolescents with idiopathic RP in relation to its persistence and potential evolution: in particular, we have tried to retrospectively define if 25-hydroxyvitamin D [25(OH)-vitamin D] could eventually predict RP outcome in the medium term and if vitamin D supplementation could mitigate RP severity.

## 2. Patients and Methods

### 2.1. Population of Patients with Idiopathic Raynaud’s Phenomenon

We retrospectively evaluated the medical records of 69 unselected Caucasian children and adolescents with idiopathic RP who were first admitted to the outpatient care unit of pediatric rheumatology at our University hospital during the period 2010–2025. Eight patients who were lost to follow-up or had insufficient/limited laboratory data were excluded from our analysis. The remaining 61 patients had undergone regular clinical and laboratory assessments to evaluate RP persistence over time and detect a possible evolution towards a connective tissue disease. The inclusion criteria for our analysis required that: (1) all patients had typical symptomatic RP lasting for at least 3 months unrelated to any previously known connective tissue disease; (2) patients’ age was less than 18 years. Exclusion criteria were: (a) the presence of established underlying diseases such as systemic sclerosis, mixed connective tissue disease or systemic lupus erythematosus at the first outpatient clinical assessment of RP; (b) previous history of any hematological disorders; (c) previous diagnosis of immunodeficiency; (d) any relationship with severe acute respiratory syndrome coronavirus 2 infection and chronic persistence of post-coronavirus infection symptoms. All patients lived in central Italy (latitude varying from 41°13′ N to 42°42′ N; ultraviolet index: 1–5 during wintertime). Neither a previous history of urticaria/allergy nor gastrointestinal disease were ever referred to by any patients or their parents/caregivers. All started presenting symptoms of RP between November and April, with the exception of one 15-year-old girl who began in late summer (September); all patients had RP affecting the hands, however heightened sensitivity to cold in the feet was reported by 23 patients of the cohort. No patients required treatment with specific anti-RP drugs as vasodilators.

The study was conducted in line with the principles of the 1964 Declaration of Helsinki and its later amendments: all patients’ parents and caregivers were informed about the aim of this project at the moment of first disease assessment, and all of them signed written consent to both unrestricted access to medical charts and the evaluation of children’s anonymized data. None of the parents declined participation in the project. The Local Ethical Committee approved the study as part of a larger protocol based on the evaluation of nutritional issues in patients with rare diseases (approval code from our Ethical Committee: 2105; approval date: 5 February 2019).

RP was evaluated using the 2-week Raynaud’s condition score (RCS), a subjective daily self-assessment measure graded on a scale of 0–10, based on the patient’s perceived impact of the frequency, duration, and severity of RP over the previous 2 weeks [7], which was assessed at baseline and at 6-month, 12-month, or further follow-up evaluations. The whole cohort was examined for a mean period of 3 ± 1.6 years.

### 2.2. Data Collection of Children and Adolescents with Idiopathic Raynaud’s Phenomenon

We evaluated demographic and general variables as sex, age at RP onset, mean duration of RP (in minutes), and the presence of other associated symptoms involving the musculo-skeletal system. Laboratory tests were assessed, including C-reactive protein (CRP), erythrosedimentation rate (ESR), serum amyloid-A (SAA), complement fractions C_3_ and C_4_, aspartate aminotransferase (AST), alanine aminotransferase (ALT), creatinine, hemoglobin, platelet count, anti-nuclear antibodies (ANA), anti-DNAds/anti-extractable nuclear antigen (ENA)/anti-centromere antibodies, lupus-like anticoagulant (LAC, measured as dilute Russell’s viper venom time (dRVVT), normally < 45″), fasting lipid parameters (total cholesterol and triglycerides), and 25(OH)-vitamin D, as potential predictors for persistence of RP and its eventual evolution over time. All participants had negative anti-transglutaminase antibodies. Serum 25(OH)-vitamin D levels were measured by automated chemiluminescence immunoassay technology; no patients were taking anti-seizure medications or vitamin D dietary supplementation at the time of blood sampling. Mean blood pressure had been assessed at the baseline visit, resulting within normal limits (for age and sex) in all. Patients completed the 2-week RCS questionnaire at baseline alongside the collection of patient’s visual analogue scale (VAS) assessments for RP-related pain and RP-related global quality of life. Among data collected, the results of nailfold videocapillaroscopy (NVC), which was performed after baseline first RP assessment, were also considered: at least 10 capillaries per finger of four fingers of both hands (from index to little finger) were examined; this assessment included: (a) the density and length of capillaries, (b) derangement of the microvascular array with avascular areas and neoangiogenesis, (c) eventual hemorrhages, (d) altered morphology of capillaries, and (e) capillary edema of different severity.

### 2.3. Statistical Analysis

Statistical analyses were performed using R (version 4.4.2; R Core Team, 2024). Continuous variables were summarized as means and standard deviations (SDs) when normally distributed, and as medians with interquartile ranges (IQRs) when distributional assumptions were unmet. Normality was assessed using the Shapiro–Wilk test. Categorical variables were expressed as frequencies and percentages. In consideration of vitamin D results, participants were stratified following vitamin D status into two groups, according to a level below or above 20 ng/mL. Between-group comparisons were performed using Student’s *t*-test for normally distributed continuous variables and the Mann–Whitney *U* test for non-normally distributed variables. Categorical variables were compared using the χ^2^ test or Fisher’s exact test, as appropriate. To assess the association between vitamin D status and study outcomes while controlling for potential confounders, multivariable logistic regression analyses were performed. The dependent variable was binary and was defined according to the cut-off of 20 ng/mL for vitamin D. Given the limited number of participants with vitamin D deficiency (*n* = 19), the multivariable analysis was prespecified to be parsimonious rather than based on data-driven univariate screening. Baseline 2-week RCS, the clinically selected variable of primary interest, was entered into a logistic regression model together with age and sex as potential confounders. The adjustment for age and sex is particularly important because both factors may influence different immune-mediated manifestations, vascular involvement, and vitamin D metabolism. To assess the robustness of the estimates to small-sample bias, a Firth penalized logistic regression was additionally performed as a sensitivity analysis. A two-sided *p*-value < 0.05 was considered statistically significant.

## 3. General Results

### 3.1. Clinical Features and Labwork Results of Children and Adolescents with Raynaud’s Phenomenon

The mean age of 61 children and adolescents enrolled in our analysis was 14 ± 4 years; 44 patients were females (corresponding to 72.1% of the whole cohort); all were followed-up in our tertiary center for an average period of 3 ± 1.6 years. Median age of RP onset was 16.0 (13.5–17.0) years; median daily duration of RP was 10.0 (5.0–15.0) minutes. Joint symptoms were present in 15/61 patients (24.6% of the cohort). The results of labwork investigations showed that inflammatory markers were generally unaltered, with a median CRP of 2.0 mg/L (0.47–3.21), a median ESR of 10.0 mm/h (7.0–14.0), and median SAA of 4.0 mg/L (4.0–8.5), overall indicating the absence of clinically relevant systemic inflammation in the study cohort. Complement levels were within the normal range, with a median C_3_ level of 110.0 mg/dL (100.0–121.0) and a median C_4_ level of 19.0 mg/dL (17.0–22.0). Liver function tests were unremarkable, with a mean AST level of 22.8 ± 8.2 IU/L and a median ALT level of 20.0 IU/L (15.0–31.0). Hematological parameters were also within normal limits, with mean hemoglobin concentration of 13.4 ± 1.2 g/dL and mean platelet count of 289.8 ± 77.2 × 10^9^/L. Renal function was preserved, as reflected by a mean serum creatinine level of 0.69 ± 0.17 mg/dL. ANA were positive in 14 out of 61 patients (22.9% of the cohort); anti-dsDNA antibodies were detected in 10 patients (16.4% of the cohort), while only one patient was positive for anti-centromere antibodies (1/61, 1.6%). No patients were positive for specific anti-ENA antibodies. LAC (measured as dRVVT) was positive in 12 patients (19.7% of the cohort). Lipid profile was within the normal ranges, with a median total cholesterol level of 106.0 mg/dL (IQR, 88.0–134.0) and a mean triglyceride level of 94.6 ± 19.8 mg/dL.

A total of 19 out of 61 patients of the cohort (31.1%) were found to have low serum 25(OH)-vitamin D (<20 ng/mL) suggesting vitamin D deficiency, whereas 3/61 patients (4.9%) had vitamin D insufficiency (with a level between 20 and 30 ng/mL). A nonspecific pattern (characterized by diffuse edema) at the NVC was observed in 19/61 (31.1%) of the overall cohort at the baseline first assessment of RP; no patients were found to have a scleroderma pattern at the NVC. A comparison of vitamin D levels with the overall results of the study is shown in Table 1.

### 3.2. Mid-Term Follow-Up of Children and Adolescents with Raynaud’s Phenomenon

All patients were followedup for an average period of 3 ± 1.6 years. Those with vitamin D deficiency at baseline received cholecalciferol supplementation (1200 IU/day during wintertime for at least 12 weeks). At the 6-month, 12-month, or further follow-up evaluations, a significant improvement of 2-week RCS was observed at 6 or 12 months in patients with initial vitamin D deficiency who received cholecalciferol supplementation [3.5 (3.0–4.0) *versus* 0.0 (0.0–1.0), *p* ˂ 0.0001]. A comparison of baseline and follow-up results of the 2-week RCS is reported in Table 2. No patients developed an overt connective tissue disease (neither systemic sclerosis nor mixed connective tissue disease and systemic lupus erythematosus), and no patients had RP complications such as fingertip ulcers during the investigation period. No patient developed any thrombotic manifestations during the same period, and LAC determination (at least after 3 months) was unrevealing in the patients who had an initial positivity.

### 3.3. Univariate Analysis

We divided patients with idiopathic RP into two subgroups according to the vitamin D cut-off of 20 ng/mL: the comparison of variables showed statistically significant differences in terms of mean daily RP duration (*p* = 0.0002), 2-week RCS at baseline (*p* = 0.0001), LAC positivity (measured as dRVVT) (*p* = 0.0007), nonspecific NVC pattern (*p* = 0.0001), pain VAS (*p* = 0.0001), and global quality of life VAS (*p* = 0.0001). Indeed, a longer duration of RP was referred by vitamin D-deficient patients compared with non-deficient ones (20.0 *versus* 9.0 min) as well as a more severe 2-week RCS (3.5 *versus* 1.5) and a more severe RP-related pain VAS (2.0 *versus* 0.0) and RP-related global quality of life VAS (1.0 *versus* 0.0).

### 3.4. Multivariable Analysis

Given the limited number of participants with vitamin D deficiency, a parsimonious multivariable logistic regression was performed including baseline 2-week RCS, age, and sex. Baseline RCS was independently associated with vitamin D deficiency (OR 3.95 per one-point increase, 95% CI 2.01–7.76, *p* < 0.001; see Table 3), whereas age and sex were not. A Firth penalized logistic regression sensitivity analysis yielded a consistent association (OR 3.44, profile-likelihood 95% CI 1.95–6.90; likelihood-ratio χ^2^ = 21.60; *p* < 0.001; see Table 4). The penalized estimate was directionally and quantitatively consistent with the conventional parsimonious logistic model, supporting robustness to small-sample bias.

## 4. Discussion

Several studies have hypothesized vitamin D deficiency as being critical in immune cell maturation as well as in the progression of many inflammatory disorders, so routine screening for vitamin D status is becoming part of standard care in pediatric health checkups. The major circulating metabolite of vitamin D, serum 25(OH)-vitamin D, is the most used biomarker for assessing vitamin D level, reflecting both total sunlight exposure and dietary calciferol intake. Low serum concentrations of 25(OH)-vitamin D have been reported in children affected by vasculitides, acute rheumatic fever, and autoinflammatory disorders, which could contribute to disease expression or complications [8,9,10,11]. RP has been also specifically reported in familial Mediterranean fever, cryopyrin-associated periodic syndrome and tumor necrosis factor receptor-associated periodic syndrome, though no standardized protocol is available to manage RP in the youngest patients [12,13,14,15]. Poor data are available to characterize the occurrence of RP in childhood and define its relationship with vitamin D status, which is classified by Endocrine Society guidelines as deficient if <20 ng/mL, insufficient if in the range of 20–29 ng/mL, or sufficient if ≥30 ng/mL [16].

We basically know that a key-issue of RP is the imbalance of vasoconstriction and vasodilation due to alterations in neural control of vascular tone and disrupted circulating mediators. However, RP is largely reported by healthy subjects without any underlying disease, though it may also work as a revelatory diagnostic clue in approximately 90% of patients with systemic sclerosis, a chronic connective tissue disease with protean severe dermatological manifestations [17].

Gupta et al. evaluated the role of vitamin D in the pathogenesis of systemic sclerosis, finding severe vitamin D deficiency (<10 ng/mL) in 34.2% of cases, but no relationship with age, sex, disease duration, positive type of autoantibodies, digital ulcerations, and visceral involvement [18]. A retrospective multi-center collection of clinical, laboratory, and treatment data of 94 Caucasian children and adolescents with RP starting at a mean age of 12.8 ± 5 years, with variable involvement of the hands, feet, and face, was performed in 2015, revealing that positive ANA found in 29% of patients could significantly predict RP progression towards a connective tissue disease [19]. Kisla Ekinci et al. determined the frequency of vitamin D deficiency in 40 pediatric patients with idiopathic RP and investigated its relationship with RP course, finding that the need for vasodilators was lower in vitamin D-deficient patients undergoing vitamin D replacement [20]. Hélou et al. performed a randomized placebo-controlled double blind study in 53 adults with RP, and more specifically in 42 vitamin D-deficient patients who were randomly assigned into either the vitamin D group (receiving a monthly supplementation of oral vitamin D, 600,000 IU during winter for 2 months) or the ‘placebo’ group; patients who received vitamin D actually had an RP self-judgment improvement after vitamin D supplementation [21].

This current study examined the association between different clinical, laboratory and instrumental data in a single-center cohort of children and adolescents with idiopathic RP who were retrospectively evaluated, following a cross-sectional baseline analysis and longitudinal follow-up. For this study, the 2-week RCS on a scale of 0–10 (reported in a paper diary) was administered to evaluate RP impact on patients’ lives. In particular, the prevalence of vitamin D deficiency at the first RP assessment and its association with RP severity were carefully evaluated.

Overall, the study cohort showed no relevant laboratory abnormalities: inflammatory markers, liver and renal function tests, hematological parameters, and lipid profile were within the normal range. The only labwork abnormality found was vitamin D deficiency in a subset of the cohort: in particular, low vitamin D levels were associated with higher 2-week RCS at baseline, suggesting a more severe clinical RP phenotype. Moreover, 19 out of 61 children and adolescents with RP showed a pattern of ‘nonspecific’ abnormalities at the NVC performed after first RP assessment (no one had a scleroderma pattern). ANA positivity did not predict any adverse outcome in 14 RP patients; it was mostly present in the four patients who also had vitamin D deficiency.

Among patients with vitamin D deficiency, cholecalciferol supplementation was associated with a statistically significant improvement in 2-week RCS at the follow-up assessments. This association may be explained by the multiple biological effects of vitamin D on vascular homeostasis and immune regulation. Indeed, vitamin D may elicit vasoprotective effects, and its deficiency has been linked to endothelial dysfunction, being characterized by reduced bioavailability of nitric oxide—a potent endothelium-dependent vasodilator—and by enhanced vasoconstrictor responses, both contributing to influence vasospasm susceptibility of RP [21]. Furthermore, inadequate vitamin D levels may promote immune dysregulation impairing different microvascular functions and exacerbating the severity of RP attacks [22].

Some preclinical studies supported the biochemical link between the genomic effect of vitamin D and regulation of endothelial function, which involves regulation of the bioavailability and bioactivity of nitric oxide [23]. However, the available evidence is insufficient to conclude the efficacy of vitamin D supplementation for improvement of endothelial function in humans, although there is some evidence of benefit with very high dose vitamin D supplementation [24]. Evidence is lacking regarding the use of lower dose supplementation for longer durations, such as one year or longer. More specifically, in our experience, following cholecalciferol supplementation, patients with baseline vitamin D deficiency experienced a significant improvement in 2-week RCS, reaching values even lower than those observed in patients with normal vitamin D levels at baseline. This finding raises the possibility that correcting vitamin D deficiency might exert beneficial effects on vascular function through improvement of endothelial function and increased nitric oxide bioavailability. Alternatively, the improved RCS observed in the vitamin D-deficient subgroup may partly reflect a larger potential for clinical improvement due to their higher baseline disease severity.

Despite these findings, several limitations of the study should be acknowledged. First, its retrospective design precludes the establishment of causal relationships. Second, the absence of a healthy control group as well as the absence of vitamin D-deficient patients untreated with vitamin D downgrades any interpretation of the association found. Consequently, although our findings would suggest the potential therapeutic benefit of cholecalciferol supplementation in vitamin D-deficient patients with idiopathic RP, they should be interpreted with extreme caution, requiring confirmation in prospective randomized controlled trials. Third, the relatively small sample size, resulting from the single-center nature of the study, may have reduced its statistical power. Fourth, data on patients’ dietary habits and outdoor activities were not available in the medical records (especially for those dating back to more than 10 years ago) and long-term clinical outcomes of RP were not systematically assessed (especially for the most recently evaluated cases), preventing a more detailed analysis. Moreover, the relatively large number of clinical, laboratory, and instrumental variables examined in this limited cohort may have increased the likelihood of chance findings due to multiple comparisons. Therefore, the statistically significant associations emerging from univariate analyses should be regarded as exploratory and interpreted with prudence. Finally, treatment allocation was not randomized and serum 25(OH)-vitamin D levels measured during the follow-up period in combination with RCS re-assessments were not systematically available. Therefore, we could not determine whether the clinical improvement paralleled biochemical correction of vitamin D deficiency, further limiting any causal interpretation of the association between cholecalciferol supplementation and RCS improvement.

Future research could integrate advanced molecular and immunological approaches, including immune phenotyping, host genetic analyses, and microbiome characterization, to better define the biological mechanisms linking vitamin D deficiency to RP in larger cohorts. A deeper understanding of RP pathways will probably improve the risk stratification process and pave the way for targeted preventive or therapeutic strategies.

## 5. Conclusions

Vitamin D deficiency is a health issue that may disproportionately affect children and adolescents, even in areas with optimal likelihood of sunlight exposure. In the present study, which provides an exploratory retrospective assessment of vitamin D status at the first clinical evaluation of pediatric RP during the period 2010–2025 in a tertiary referral center, 19 out of 61 patients (31.1%) had serum 25(OH)-vitamin D levels below 20 ng/mL. All patients were evaluated with 2-week RCS, a highly reproducible and broadly applicable measure that is sensitive to assessing RP impact on patients’ everyday lives, for a mean investigation period of 3 ± 1.6 years. Vitamin D deficiency found at the first RP evaluation was independently associated with greater disease severity, as reflected by significantly higher baseline RCS at the parsimonious multivariable logistic regression with correction for confounders. Importantly, post-supplementation 2-week RCS in vitamin D-deficient patients with RP significantly improved at the following assessments. However, the limitations of this study do not allow one to establish a causal relationship for vitamin D deficiency, and the improvement found may reflect the biological effects of vitamin supplementation, a naturally benign course of the disease, or regression toward the mean.

Taken together, our findings support the need for integrated rheumatologic management and routine assessment of vitamin D status, particularly in symptomatic children and adolescents with idiopathic RP or in those with recognized risk factors for vitamin D deficiency, and suggest that correction of vitamin D deficiency could be considered a potentially beneficial adjunctive strategy. However, adequately powered prospective randomized controlled trials will help to determine whether vitamin D supplementation may directly improve disease severity, to identify the patients most likely to benefit, and to clarify the mechanisms linking vitamin D deficiency to the pathophysiology of RP.

## Figures and Tables

**Table 1:** Baseline characteristics of the study cohort compared between groups according to 25(OH)-vitamin D level (cut-off of 20 ng/mL).

*Variable*	Overall Cohort (*n* = 61)	25(OH)-D < 20 ng/mL (*n* = 19)	25(OH)-D ≥ 20 ng/mL (*n* = 42)	*p*-Value
**Demographic and clinical characteristics**				
Female sex, *n* (%)	44 (72.1)	16 (84.2)	28 (66.7)	0.220
Age at RP onset, years	16.0 (13.5–17.0)	16.0 (12.0–17.0)	15.0 (14.0–17.0)	0.990
Mean daily RP duration, minutes	10.0 (5.0–15.0)	20.0 (11.0–21.0)	9.0 (5.0–12.0)	**0.0002**
2-week Raynaud’s Condition Score at baseline	2.0 (1.0–3.0)	3.5 (3.0–4.0)	1.5 (1.0–2.0)	**0.0001**
Arthralgias, *n* (%)	15 (24.6)	6 (31.6)	9 (21.4)	0.600
Pain VAS	0.0 (0.0–1.0)	2.0 (0.5–2.5)	0.0 (0.0–0.5)	**0.0001**
Global quality of life VAS	0.0 (0.0–1.0)	1.0 (0.0–1.5)	0.0 (0.0–0.0)	**0.0001**
**Laboratory findings**				
CRP, mg/L	2.0 (0.47–3.21)	1.40 (0.30–2.96)	2.40 (0.50–3.22)	0.180
ESR, mm/h	10.0 (7.0–14.0)	11.0 (7.0–15.5)	10.0 (7.0–13.8)	0.490
SAA, mg/L	4.0 (4.0–8.5)	4.0 (3.5–9.8)	4.3 (4.0–8.4)	0.870
C_3_, mg/dL	110.0 (100.0–121.0)	107.0 (96.5–119.0)	110.5 (100.3–123.3)	0.620
C_4_, mg/dL	19.0 (17.0–22.0)	19.0 (15.5–20.5)	19.0 (17.0–22.0)	0.430
AST, IU/L	22.8 ± 8.2	23.3 ± 10.0	22.6 ± 7.3	0.760
ALT, IU/L	20.0 (15.0–31.0)	20.0 (13.0–33.5)	22.5 (15.3–28.8)	0.990
Hemoglobin, g/dL	13.4 ± 1.2	13.1 ± 1.0	13.6 ± 1.2	0.160
Platelet count, ×10^9^/L	289.8 ± 77.2	290.8 ± 67.3	289.3 ± 82.0	0.940
Creatinine, mg/dL	0.69 ± 0.17	0.72 ± 0.19	0.67 ± 0.15	0.350
LAC positivity, *n* (%)	12 (19.7)	9 (47.4)	3 (7.1)	**<0.001**
Patients with positive anti-nuclear antibodies, *n* (%)	14 (22.9)	4 (21.0)	10 (23.8)	1.0
Patients with positive anti-dsDNA antibodies, *n* (%)	10 (16.4)	3 (15.8)	7 (16.7)	1.0
Patients with positive anti-centromere antibodies, *n* (%)	1 (1.6)	1 (5.3)	0 (0.0)	0.310
Cholesterol, mg/dL	106.0 (88.0–134.0)	110.0 (88.5–158.0)	105.0 (89.0–127.0)	0.450
Triglycerides, mg/dL	94.6 ± 19.8	87.9 ± 19.0	97.7 ± 19.6	0.070
**Nailfold videocapillaroscopy**				
NVC nonspecific pattern, *n* (%)	19 (31.1)	13 (68.4)	6 (14.3)	**<0.001**

Data are reported as median (IQR) for non-normally distributed variables, mean ± SD for normally distributed variables, and *n* (%) for categorical variables. Normality was assessed using the Shapiro–Wilk test. Comparisons were performed using the Mann–Whitney U test, Student’s *t*-test, χ^2^ test, or Fisher’s exact test, as appropriate. Statistically significant *p*-values are shown in bold. *Abbreviations:* 25(OH)-D, 25-hydroxyvitamin D; RP, Raynaud’s phenomenon; VAS, visual analogue scale; CRP, C-reactive protein; ESR, erythrosedimentation rate; SAA, serum amyloid-A; LAC, lupus-like anticoagulant; NVC, nailfold videocapillaroscopy.

**Table 2:** Raynaud’s Condition Score (RCS) at baseline and at the follow-up of patients with and without vitamin D deficiency.

	2-Week RCS at Baseline	2-Week RCS at the Follow-Up	*p*
Patients with vitamin D level ≥ 20 ng/mL	1.5 (1.0–2.0)	1.0 (0.0–1.0)	ns
Patients with vitamin D level ˂ 20 ng/mL	3.5 (3.0–4.0)	0 (0.0–1.0)	0.0001

**Table 3:** Parsimonious multivariable logistic regression analysis adjusted for sex and age comparing groups according to the level of 25(OH)-vitamin D (cut-off of 20 ng/mL). Statistically significant *p*-values (<0.05) are shown in bold.

*Variable*	OR	95% CI	*p*-Value
Female sex	1.253	0.238–6.603	0.791
Age at first RP onset (years)	0.879	0.630–1.228	0.450
2-week Raynaud’s Condition Score at baseline	3.946	2.007–7.757	<0.001

*Abbreviations:* OR, odds ratio; CI, confidence interval; RP: Raynaud’s phenomenon.

**Table 4:** Penalized logistic regression sensitivity analysis; profile-likelihood confidence intervals are shown for the primary predictor.

*Variable*	Firth Penalized OR	Profile-Likelihood 95% CI	*Likelihood-Ratio* (χ^2^)	*p*-Value
2-week Raynaud’s Condition Score at baseline	3.439	1.952–6.904	21.60	<0.001
Age at first RP onset, years	0.896	—		
Female sex	1.169	—		

*Abbreviations:* OR, odds ratio; CI, confidence interval; RP: Raynaud’s phenomenon.

## Data Availability

Anonymized data related to this study are available upon reasonable request.

## Abbreviations

The following abbreviations are used in this manuscript:

ALT	alanine aminotransferase
ANA	anti-nuclear antibodies
AST	aspartate aminotransferase
anti-ENA	anti-extractable nuclear antigen
CRP	C-reactive protein
dRVVT	dilute Russell’s viper venom time
ESR	erythrosedimentation rate
LAC	lupus-like anticoagulant
NVC	nailfold videocapillaroscopy
RCS	Raynaud condition score
RP	Raynaud’s phenomenon
SAA	serum amyloid-A
VAS	visual analogue scale
25(OH)-vitamin D	25-hydroxyvitamin D

## References

[1] Herrick A.L., Wigley F.M. (2020). Raynaud’s phenomenon. Best Pract. Res. Clin. Rheumatol..

[2] Maciejewska M., Sikora M., Maciejewski C., Alda-Malicka R., Czuwara J., Rudnicka L. (2022). Raynaud’s phenomenon with focus on systemic sclerosis. J. Clin. Med..

[3] Valladales-Restrepo L.F., Henao-Martínez M., Velasquez-Quimara S., Giraldo-Galeano S., Saldarriaga-Rivera L.M., Machado-Alba J.E. (2025). Sociodemographic and pharmacological characteristics of patients with primary and secondary Raynaud’s phenomenon. A cross-sectional study. Vasc. Dis..

[4] Hughes M., Snapir A., Wilkinson J., Snapir D., Wigley F.M., Herrick A.L. (2015). Prediction and impact of attacks of Raynaud’s phenomenon, as judged by patient perception. Rheumatology.

[5] Rigante D., Fastiggi M., Ricci F., D’Errico F., Bracci B., Guerriero C. (2017). Handy hints about Raynaud’s phenomenon in children: A critical review. Pediatr. Dermatol..

[6] Khouri C., Lepelley M., Bailly S., Blaise S., Herrick A.L., Matucci-Cerinic M., Allanore Y., Trinquart L., Cracowski J.L., Roustit M. (2019). Comparative efficacy and safety of treatments for secondary Raynaud’s phenomenon: A systematic review and network meta-analysis of randomised trials. Lancet Rheumatol..

[7] Merkel P.A., Herlyn K., Martin R.W., Anderson J.J., Mayes M.D., Bell P., Korn J.H., Simms R.W., Csuka M.E., Medsger T.A. (2002). Scleroderma Clinical Trials Consortium. Measuring disease activity and functional status in patients with scleroderma and Raynaud’s phenomenon. Arthritis Rheum..

[8] Wang J., Ye W.P. (2023). The comparisons of vitamin D3 levels in IgA vasculitis across different subgroups and healthy children: A comparative study. Transl. Pediatr..

[9] Yang D.D., Zeng Y.Y. (2026). Vitamin D and Kawasaki disease. Front. Pharmacol..

[10] Tetik Dinçer B., Akboğa E., Melikoğlu F., Özçelik G. (2026). Association between vitamin D, vitamin B12 and folate levels and persistent subclinical inflammation in pediatric familial Mediterranean fever. Children.

[11] Onan S.H., Demirbilek H., Aldudak B., Bilici M., Demir F., Yılmazer M.M. (2017). Evaluation of vitamin D levels in patients with acute rheumatic fever. Anatol. J. Cardiol..

[12] Dinç A., Melikoğlu M., Korkmaz C., Fresko I., Ozdoğan H., Yazici H. (2001). Nailfold capillary abnormalities in patients with familial Mediterranean fever. Clin. Exp. Rheumatol..

[13] Cantarini L., Lucherini O.M., Frediani B., Brizi M.G., Bartolomei B., Cimaz R., Galeazzi M., Rigante D. (2011). Bridging the gap between the clinician and the patient with cryopyrin-associated periodic syndromes. Int. J. Immunopathol. Pharmacol..

[14] Rigante D., Lopalco G., Vitale A., Lucherini O.M., De Clemente C., Caso F., Emmi G., Costa L., Silvestri E., Andreozzi L. (2014). Key facts and hot spots on tumor necrosis factor receptor-associated periodic syndrome. Clin. Rheumatol..

[15] Vitale A., Rigante D., Lucherini O.M., Caso F., Muscari I., Magnotti F., Brizi M.G., Guerrini S., Patti M., Punzi L. (2013). Biological treatments: New weapons in the management of monogenic autoinflammatory disorders. Mediat. Inflamm..

[16] Holick M.F., Binkley N.C., Bischoff-Ferrari H.A., Gordon C.M., Hanley D.A., Heaney R.P., Murad M.H., Weaver C.M. (2011). Evaluation, treatment, and prevention of vitamin D deficiency: An Endocrine Society clinical practice guideline. J. Clin. Endocrinol. Metab..

[17] Viswanath V., Phiske M.M., Gopalani V.V. (2013). Systemic sclerosis: Current concepts in pathogenesis and therapeutic aspects of dermatological manifestations. Indian J. Dermatol..

[18] Gupta S., Mahajan V.K., Yadav R.S., Mehta K.S., Bhushan S., Chauhan P.S., Rawat R., Sharma V. (2018). Evaluation of serum vitamin D levels in patients with systemic sclerosis and healthy controls: Results of a pilot study. Indian Dermatol. Online J..

[19] Falcini F., Rigante D., Candelli M., Martini G., Corona F., Petaccia A., La Torre F., Raffaele C.G., Matucci Cerinic M. (2015). Anti-nuclear antibodies as predictor of outcome in a multi-center cohort of Italian children and adolescents with Raynaud’s phenomenon. Clin. Rheumatol..

[20] Kisla Ekinci R.M., Taskin Karacay I.E., Celik U. (2022). Serum vitamin B12 and D levels in children with primary Raynaud phenomenon: A retrospective cohort study. Eur. J. Clin. Nutr..

[21] Kim D.H., Meza C.A., Clarke H., Kim J.S., Hickner R.C. (2020). Vitamin D and endothelial function. Nutrients.

[22] Hélou J., Moutran R., Maatouk I., Haddad F. (2013). Raynaud’s phenomenon and vitamin D. Rheumatol. Int..

[23] Zhang Q., Zhang M., Wang H., Sun C., Feng Y., Zhu W., Cao D., Shao Q., Li N., Xia Y. (2018). Vitamin D supplementation improves endothelial dysfunction in patients with non-dialysis chronic kidney disease. Int. Urol. Nephrol..

[24] Stricker H., Tosi Bianda F., Guidicelli-Nicolosi S., Limoni C., Colucci G. (2012). Effect of a single, oral, high-dose vitamin D supplementation on endothelial function in patients with peripheral arterial disease: A randomised controlled pilot study. Eur. J. Vasc. Endovasc. Surg..

